# Oriental herbal medicine for insomnia in the elderly with hypertension: A systematic review protocol

**DOI:** 10.1097/MD.0000000000012200

**Published:** 2018-09-07

**Authors:** Chan-Young Kwon, Boram Lee, Sun-Yong Chung, Jong Woo Kim, Sang-Ho Kim

**Affiliations:** a Department of Clinical Korean Medicine, Graduate School, Kyung Hee University; b Department of Korean Medicine, Kyung Hee University Korean Medicine Hospital at Gangdong, Seoul; c Department of Neuropsychiatry of Korean Medicine, Pohang Korean Medicine Hospital, Daegu Haany University, Pohang-si, Gyeongsangbuk-do, Republic of Korea.

**Keywords:** herbal medicine, hypertension, insomnia, protocol, systematic review

## Abstract

**Background:**

Hypertension and insomnia are common diseases in the elderly. Oriental herbal medicine has been widely used in East Asia, and it has been considered as a promising alternative to conventional pharmacotherapy because of its efficacy in geriatrics. Moreover, since oriental herbal medicine acts on multiple targets, it may affect both hypertension and insomnia at the same time, which can reduce the risk of polypharmacy in this population. This systematic review will assess the efficacy and safety of oriental herbal medicine in the elderly with both insomnia and hypertension.

**Methods:**

Thirteen databases including English, Chinese, Korean, and Japanese databases will be searched from their inception to August 2018. We will include randomized controlled trials assessing oriental herbal medicine for insomnia in the elderly with hypertension. The methodological quality of the included studies will be evaluated using the Cochrane Collaboration's risk of bias tool, and confidence in the cumulative evidence will be evaluated using the Grading of Recommendations Assessment, Development, and Evaluation instrument.

**Results:**

This review will provide evidence to determine the efficacy and safety of oriental herbal medicine in the elderly with both insomnia and hypertension.

**Conclusions:**

Our results will help clinicians and health policy makers take informed decisions regarding the use of oriental herbal medicine in the elderly. It will also provide evidence-based oriental herbal medicine data for elderly patients suffering from multiple diseases and their families.

## 1 Introduction

Insomnia and hypertension are both common in the elderly and have a wide range of effects on the overall health status of this population. Insomnia is defined as “a persistent difficulty with sleep initiation, duration, consolidation, or quality that occurs despite adequate opportunity and circumstances for sleep, and results in some form of daytime impairment” in the International Classification of Sleep Disorders (ICSD), Third Edition.^[1]^ According to published epidemiological studies, the prevalence of insomnia in the elderly population ranges from 9% to 34%.^[2–4]^

Insomnia, in itself, not only causes problems such as a serious impairment of quality of life, but also increases the risks of morbidity and mortality. For example, the associations between insomnia and increased risk of memory impairment,^[5]^ dementia,^[6]^ depression,^[7]^ and cardiovascular disease^[8]^ have already been reported. According to a long-term community-based cohort study, persistent insomnia is associated with increased risks of all-cause and cardiopulmonary mortality.^[9]^ In addition, some studies have reported that certain insomnia symptoms such as difficulty initiating sleep and nonrestorative sleep are associated with an increased risk of mortality.^[10]^ To treat this disorder, nonpharmacological interventions such as cognitive behavior therapy for insomnia and pharmacological interventions such as benzodiazepines, zaleplon, zolpidem, and zopiclone have been used.^[11]^

Stage 1 hypertension is defined as “systolic blood pressure between 130 and 139 mmHg or diastolic blood pressure between 80 and 89 mmHg” in a recent clinical practice guideline of the American College of Cardiology/American Heart Association.^[12]^ That is, the state of blood pressure of ≥130/80 mmHg is defined as hypertension. The prevalence of hypertension in the elderly has been extensively studied. The prevalence varied from 40% to 75% according to the country, region, year, and race, and it has increased over time.^[13–15]^

Hypertension is not only a well-known cardiovascular risk factor,^[16]^ but also related with an increased risk of morbidity, including metabolic disorders^[17]^ and frailty.^[18]^ In a recent large-scale cohort study, uncontrolled hypertension was also associated with increased risks of all-cause and cardiovascular disease mortality.^[19]^ Treatments of hypertension usually involve the use of antihypertensives and lifestyle modifications including weight loss, Dietary Approaches to Stop Hypertension (DASH) diet, sodium reduction, and increased physical activity.^[12]^

Recently, studies on the relationship between these 2 diseases have been reported. In a systematic review of 64 studies, a strong correlation was found between insomnia and hypertension.^[20]^ To explain this association, a biological model of the dysfunction of hypothalamic-pituitary-adrenal axis has been proposed, but the mechanism is still unclear.^[21]^ However, psychological factors, including emotional distress, and biological factors, including excessive activity of arousal-related neural pathways, may be involved.^[21]^

In conventional medicine, the use of hypnotics or sleep inducers and antihypertensives in elderly patients suffering from both insomnia and hypertension is common. However, because benzodiazepines and other sedative-hypnotic drugs are frequently associated with adverse outcomes including their anticholinergic effects and increased risks of falls and hip fractures in the elderly,^[22]^ a safer and more effective treatment is needed. More importantly, the issue of polypharmacy in the elderly population was highlighted recently.^[23]^ Polypharmacy is described as taking ≥5 medications daily.^[24]^ One survey found that >50% of women Medicare beneficiaries took ≥5 medications daily, with 12% taking ≥10 medications daily.^[25]^ According to a recent British birth cohort study, polypharmacy at age 60 to 64 and age 69 was associated with poorer physical and cognitive capability, even after adjusting for disease burden.^[26]^ There is a great opportunity to reduce the number of medications taken by patients, with the potential to reduce both side effects and drug interactions. Therefore, the importance of reducing the types of medications for the elderly, including hypertensives, should be emphasized.

Oriental herbal medicine (OHM) is one of the traditional medicine treatment modalities used mainly in East Asia and has been used for thousands of years in the treatment of various diseases. Some OHMs have already been reviewed for their efficacy and safety in the treatment of insomnia and hypertension. Recently, OHM has been considered as a promising alternative to pharmacotherapy based on its efficacy in geriatrics.^[27,28]^ In addition, since OHMs act on multiple targets,^[29]^ an OHM can potentially affect both hypertension and insomnia at the same time, which means that the risk of polypharmacy and its complications may reduce.

Therefore, this review will comprehensively assess the therapeutic efficacy and safety of OHM in the elderly with insomnia and hypertension, and will discuss the possibility of OHM in terms of the polypharmacy problem.

## 2 Methods

### 2.1 Study registration

The protocol for this systematic review has been registered in the International Prospective Register of Systematic Reviews, PROSPERO with registration number (CRD42018104095) in August 6, 2018 (Available at: https://www.crd.york.ac.uk/prospero/display_record.php?RecordID=104095). We will conduct a systematic review according to this protocol, but if protocol amendments occur, the dates, changes, and rationales for each amendment will be tracked in PROSPERO. We report this protocol according to the Preferred Reporting Items for Systematic Review and Meta-Analysis Protocols (PRISMA-P) 2015 statement,^[30]^ and the Cochrane Handbook for Systematic Reviews of Interventions.^[31]^

### 2.2 Ethical approval

Ethical approval is not required because this protocol is for a systematic review, not for a clinical study.

### 2.3 Data sources and search strategy

The following databases will be systematically searched from their inception to August 2018 by 2 independent researchers (CYK and BL): Medline via PubMed, EMBASE via Elsevier, the Cochrane Central Register of Controlled Trials [CENTRAL], Allied and Complementary Medicine Database [AMED] via EBSCO, Cumulative Index to Nursing and Allied Health Literature [CINAHL] via EBSCO, and PsycARTICLES via ProQuest. We will also search 3 Korean databases (Oriental Medicine Advanced Searching Integrated System [OASIS], Research Information Service System [RISS], and Korea Citation Index [KCI]), 3 Chinese databases (China National Knowledge Infrastructure [CNKI], Wanfang Data, and VIP), and 1 Japanese database (CiNii). In addition, the reference lists of the relevant articles will be checked, and a manual search of Google Scholar will be conducted to identify additional trials. We will include not only the literature published in journals, but also gray literature, such as theses and conference proceedings. The search strategy for the Medline database is shown in Table 1, which will be suitably modified and used for the other databases.

**Table 1:**
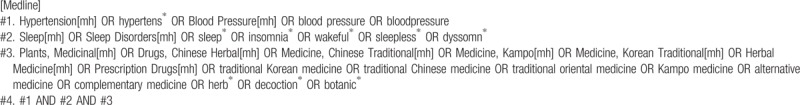
Search strategy for the Medline database.

### 2.4 Inclusion and exclusion criteria

#### 2.4.1 Types of studies

We will include randomized controlled trials (RCTs) that evaluate the efficacy of OHM in the elderly with both insomnia and hypertension. We will exclude quasi-RCTs using quasi-random methods such as alternate allocation or allocation by birth date. If the report uses the word “randomization (
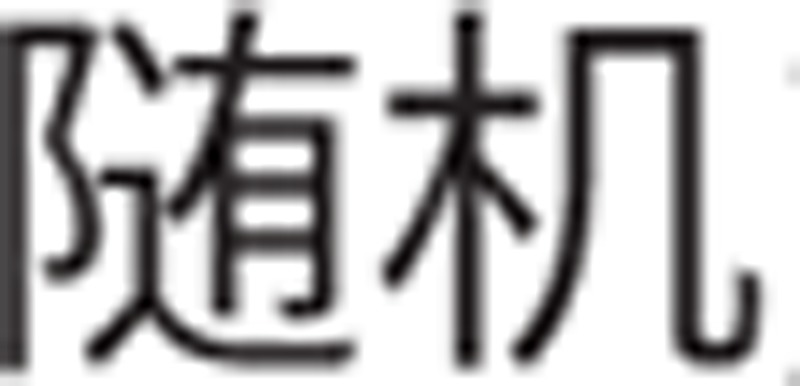
)” but the detailed method is not mentioned, it will be regarded as a RCT and included in this review. However, selection bias assessed with a random sequence generation method will be evaluated as high risk. We will include both parallel and crossover studies. In crossover studies, only the first phase data will be used in calculating the effect size and conducting meta-analysis. There will be no language restriction.

#### 2.4.2 Types of participants

This study will include RCTs on patients with a diagnosis of both hypertension and insomnia, using standardized diagnostic tools, assessment tools, textbook, or clinical symptoms. Studies that do not provide diagnostic criteria for hypertension and insomnia in participants will be excluded. We will include studies on elderly people with an average age of 60 years or older. There will be no restriction on sex or race of participants. Studies will be excluded if participants have serious systemic disease, congenital disease, cognitive dysfunction, or drug allergies.

#### 2.4.3 Types of interventions

We will include only those studies using the OHM prescribed based on traditional East Asian medicine theories as experimental intervention. Studies on OHM of any formulation (e.g., decoction, tablets, capsules, pills, powders, and extracts) as experimental interventions will be included. Studies involving OHM combined with other therapies as experimental interventions will be included if the other therapies are used equally in both the experimental and the control groups. Except for patent drugs, studies that do not list the composition of the OHM used will be excluded. Studies comparing different types of OHM will be excluded. As control interventions, we will include Western medication, placebo, or no treatment. No other restrictions will be placed on the control interventions.

#### 2.4.4 Types of outcome measurements

Primary outcomes:

(1) Change in the degree of insomnia as measured by validated assessment tools, such as the Pittsburgh Sleep Quality Index^[32]^ and the Insomnia Severity Index^[33]^
(2) Change in drug use rate

### 2.5 Secondary outcomes:

(1) Change in the blood pressure.
(2) Change in the mental health as measured by validated assessment tools, such as the Hamilton Anxiety Rating Scale,^[34]^ the Hamilton Depression Rating Scale,^[35]^ and the Geriatric Depression Scale.^[36]^
(3) Total effective rate.

Total effective rate is a non-validated outcome measure that is secondarily processed according to certain evaluation criteria, such as clinical symptom improvement or the improvement rate of other quantified outcomes. In the assessment of the total effective rate, the participants are generally classified as “cured (
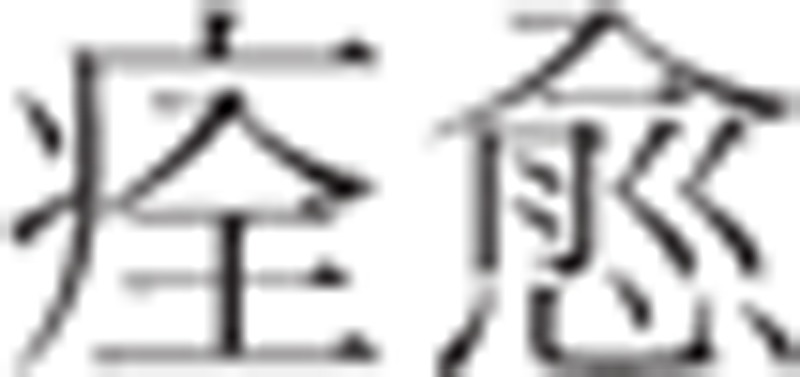
),” “markedly improved (
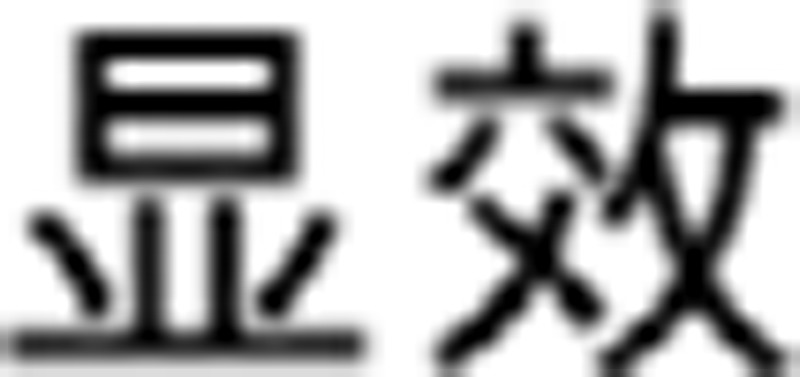
),” “improved (
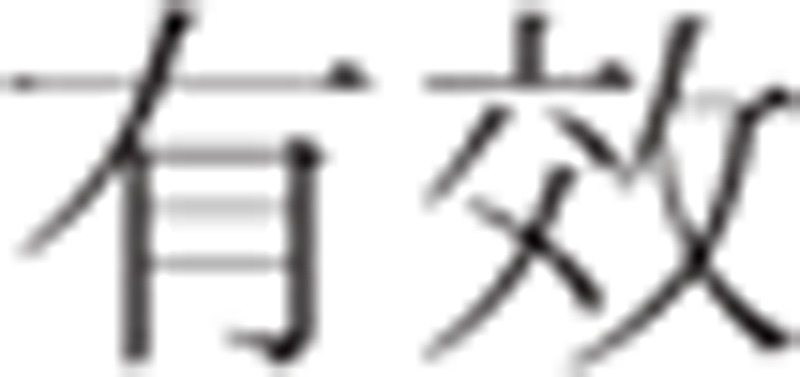
),” or “non-responder (
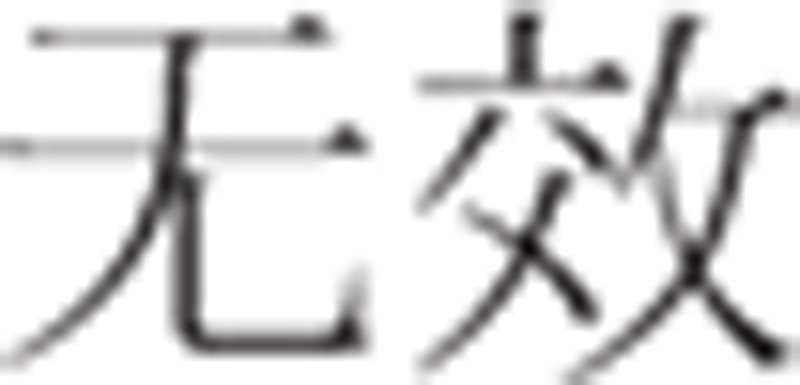
)” after the treatment. The total effective rate is calculated consistently using the following formula:

Total effective rate = N1 + N2 + N3/*N*, where N1, N2, N3, and *N* are the number of patients who are cured, markedly improved, improved, and who comprise the sample size, respectively.

(4) Adverse events as measured by the Treatment Emergent Symptom Scale^[37]^ or the incidence.

### 2.6 Study selection

Two independent researchers (CYK and BL) will conduct the study selection process according to the above inclusion criteria. After removing duplicates, we will evaluate the titles and abstracts of the searched studies for first inclusion. Then, we will evaluate the full-texts of the remaining articles for final inclusion. Any disagreement on study selection will be resolved through discussion with other researchers. Quotations of included articles will be made available online to researchers using Zotero (Roy Rosenzweig Center for History and New Media at George Mason University, VA), a reference management software program. The literature selection process will be reported in accordance with the Preferred Reporting Items for Systematic Reviews and Meta-Analyses (PRISMA) guidelines.^[38]^

### 2.7 Data extraction

Using a standardized data collection form, 2 independent researchers (CYK and BL) will perform and cross-check the data extraction. Discrepancies will be resolved through discussion with other researchers. The extracted items will include the first author's name, year of publication, country, sample size and dropout, details of participants, experimental intervention, and comparison, duration of intervention, main outcome measures, and adverse events.

The extracted data will be recorded using Excel 2010 (Microsoft, Redmond, WA), and will be shared among researchers in Dropbox (Dropbox, Inc., CA) folders.

### 2.8 Quality assessment

Two independent researchers (CYK and BL) will assess methodological quality of the included studies and the quality of evidence for each main finding. Discrepancies will be resolved through discussion with other researchers.

The methodological quality of the included RCTs will be assessed using the Cochrane Collaboration's risk of bias tool.^[39]^ The following items will be assessed: random sequence generation, allocation concealment, blinding of participants and personnel, blinding of outcome assessment, completeness of outcome data, selective reporting, and other sources of bias. Each item will be evaluated and categorized into 3 groups: “low risk,” “unclear,” or “high risk.”

We will use the Grading of Recommendations Assessment, Development, and Evaluation (GRADE) approach to evaluate the quality of evidence for each main finding.^[40]^ We will assess risk of bias, inconsistency, indirectness, imprecision of results, and probability of publication bias using a four-part scale (“very low,” “low,” “moderate,” or “high”). The results of each evaluation will be presented through the Summary of Findings table. The evaluation process will be shared among researchers using the online program GRADEpro (https://gradepro.org/).

### 2.9 Data synthesis and analysis

Descriptive analysis of the details of participants, interventions, and outcomes will be conducted for all included studies. Quantitative synthesis will be performed if there are studies using the same type of experimental intervention, comparison, and outcome measure. The data will be pooled as a mean difference or a standardized mean difference with 95% confidence intervals (CIs) for continuous outcomes and as a risk ratio with 95% CIs for dichotomous outcomes.

Heterogeneity between the studies in terms of effect measures will be assessed using both the Chi-squared test and the I-squared statistic. We will consider I-squared values >50% and 75% indicative of substantial heterogeneity and considerable heterogeneity, respectively. In the meta-analyses, a random effects model will be used when the heterogeneity is significant, while a fixed-effect model will be used when the heterogeneity is not significant. The fixed-effect model will be also used when the number of studies included in a meta-analysis is very small, where the estimates of inter-study variance have poor accuracy.^[41]^

All statistical analyses will be conducted using the Cochrane Collaboration's software program Review Manager (RevMan) version 5.3 for windows (Copenhagen, The Nordic Cochrane Centre, the Cochrane Collaboration, 2012), and will be shared among researchers in Dropbox (Dropbox, Inc., CA) folders. We will contact corresponding authors of the included studies via email to request additional information if the data are insufficient or ambiguous.

#### 2.9.1 Subgroup analysis

If heterogeneity is evaluated as considerable (I-squared value >75%) and the necessary data are available, we will conduct a subgroup analysis to explore the possible cause of the heterogeneity. Subgroup analysis will be conducted according to the following criteria: the disease severity and the treatment period.

#### 2.9.2 Assessment of reporting biases

If >10 trials included in the analysis are available, we will assess reporting biases such as publication bias using funnel plots. When there is an asymmetry of the funnel plot, we will try to explain the possible reasons.

## 3 Discussion

In East Asia, OHM has been used for thousands of years to treat various diseases. This treatment modality has recently been considered as an alternative to conventional pharmacotherapy in the elderly,^[27,28]^ and is particularly promising for patients with multiple pathologic conditions due to its ability to act on multiple targets.^[29]^ However, there has been no systematic review investigating the safety and efficacy of OHM in the elderly with both hypertension and insomnia, which are common conditions in this population. Therefore, we will comprehensively collect the available evidence to systematically and critically review the safety and efficacy of OHM in this regard. The significance of OHM for the polypharmacy problem in the elderly will also be examined. Our results will help clinicians and health policy makers take informed decisions regarding the use of OHM in the elderly. It will also provide evidence-based OHM data for elderly patients suffering from multiple diseases and their families.

## Author contributions

The study was conceptualized by CYK. The search strategy was developed by CYK and BL. CYK and BL drafted the protocol. SYC, JWK, and SHK revised the manuscript. SHK submitted the manuscript for publication. All authors have read and approved the final manuscript.

**Conceptualization:** Chan-Young Kwon.

**Methodology:** Chan-Young Kwon, Boram Lee.

**Writing – original draft:** Chan-Young Kwon, Boram Lee.

**Writing – review & editing:** Sun-Yong Chung, Jong Woo Kim, Sang-Ho Kim.

**Supervision:** Sang-Ho Kim.

Chan-Young Kwon: 0000-0003-0068-9904.
